# Long-term individualized monitoring of sympatric bat species reveals distinct species- and demographic differences in hibernation phenology

**DOI:** 10.1186/s12862-022-01962-6

**Published:** 2022-01-28

**Authors:** Frauke Meier, Leo Grosche, Christine Reusch, Volker Runkel, Jaap van Schaik, Gerald Kerth

**Affiliations:** 1 Zoological Institute and Museum, Applied Zoology and Nature Conservation, Greifswald University, Greifswald, Germany; 2 BVF Bundesverband für Fledermauskunde Deutschland e.V., Erfurt, Germany; 3 Present Address: Department of Evolutionary Ecology, Leibniz Institute for Zoo and Wildlife Research, Berlin, Germany

**Keywords:** Daubenton’s bats, *Myotis daubentonii*, Natterer’s bats, *Myotis nattereri*, Chiroptera, Hibernation timing, Hibernation phenology

## Abstract

**Background:**

Hibernation allows species to conserve energy and thereby bridge unfavorable environmental conditions. At the same time, hibernation imposes substantial ecological and physiological costs. Understanding how hibernation timing differs within and between species can provide insights into the underlying drivers of this trade-off. However, this requires individualized long-term data that are often unavailable. Here, we used automatic monitoring techniques and a reproducible analysis pipeline to assess the individualized hibernation phenology of two sympatric bat species. Our study is based on data of more than 1100 RFID-tagged Daubenton’s bats (*Myotis daubentonii*) and Natterer’s bats (*Myotis nattereri*) collected over seven years at a hibernaculum in Germany. We used linear mixed models to analyze species-, sex- and age-specific differences in entrance, emergence and duration of the longest continuous period spent in the hibernaculum.

**Results:**

Overall, Daubenton’s bats entered the hibernaculum earlier and emerged later than Natterer’s bats, resulting in a nearly twice as long hibernation duration. In both species, adult females entered earlier and emerged from hibernation later than adult males. Hibernation duration was shorter for juveniles than adults with the exception of adult male Natterer’s bats whose hibernation duration was shortest of all classes. Finally, hibernation timing differed among years, but yearly variations in entrance and emergence timing were not equally shifted in both species.

**Conclusions:**

Our results suggest that even in sympatric species, and across sex and age classes, hibernation timing may be differentially affected by environmental conditions. This highlights the necessity of using individualized information when studying the impact of changing environments on hibernation phenology.

**Supplementary Information:**

The online version contains supplementary material available at 10.1186/s12862-022-01962-6.

## Background

Many mammal species, and at least one bird species, are able to substantially reduce their metabolism during unfavorable conditions by means of hibernation [1]. Hibernation constitutes a trade-off between energy conservation during periods of unfavorable conditions (i.e., low temperature and/or reduced prey availability) and ecological and physiological costs of metabolic depression, including susceptibility to predation, reduced immune function, and accumulation of metabolic wastes [2]. During the hibernation phase, energy conservation and consumption are a function of the length and depth of torpor bouts and their relative energy savings compared to euthermy [3]. In recent years, a growing body of literature has highlighted how animals are able to influence energy expenditure through changes in arousal rate [4, 5] and the selection of particular microclimatic [6, 7] as well as social conditions (i.e., clustering) [8, 9]. Crucially, however, in fat-storing hibernators, the overall amount of energy available is fundamentally limited by the amount of body fat an individual is able to accrue prior to hibernation [10]. In addition, remaining energy reserves and food availability upon emergence from hibernation may strongly shape reproductive ability, and thus fitness (e.g. [11]). Therefore, hibernation phenology, i.e., the overall length and timing of hibernation [12], is a key component to understanding the energy balance of the hibernation trade-off.

The timing of mammalian hibernation in the temperate zone is influenced by interacting biotic and abiotic factors [13, 14], and can be highly species-specific. For example, differences in foraging ecology between species can lead to large differences in food availability before, during, and after the hibernation season via differences in prey phenology [15], thereby allowing some species to remain active longer and emerge earlier. Similarly, species may also differ in their ability to plastically adjust their hibernation timing based on annual weather variation and prey availability, or their ability to adapt and evolve in response to persistent directional changes such as climate change [16].

Within species, hibernation timing can vary intrinsically, based on characteristics such as sex and age. The seasonal timing of reproductive investment may differ strongly between sexes [17, 18], especially in bats where there is a clear desynchronization of reproductive investment [19]. In male bats, spermatogenesis and reproductive effort occur from late summer into the hibernation phase, whereas female bats face highest reproductive costs directly after hibernation, at the onset of gestation [20]. As a result, males face pressure to build up sufficient energy reserves prior to hibernation but do not need to invest heavily in reproduction upon emergence [21], while for females, condition upon emergence and energy savings during the hibernation season may directly affect reproductive success. This asymmetry has been observed in *Myotis lucifugus*, where sex differences were observed in arousal rate and torpor bout magnitude, resulting in a slower decline in body condition in females than males (‘thrifty female hypothesis’ [22, 23]). Notably, it also affects overall hibernation timing in this species, with males remaining active longer but females emerging earlier from hibernation, presumably due to the advantages of early parturition [19]. In addition to sex differences, hibernation phenology may also differ strongly between juveniles and adults. Juvenile bats have been observed to have lower fat reserves [21] and arrive at hibernacula later than adults [24], possibly due to high energy investment in growth.

In bats, outside of the detailed observations for *M. lucifugus* discussed above, information on hibernation timing at the individual or even demographic level is fragmented and largely lacking. Faced with strongly declining insect populations [25–27] and the increasing frequency of extreme weather events and climate change, detailed species and demographic insights into the hibernation behavior of temperate zone bats are urgently needed.

Automated registration of RFID-tagged individuals at the entrance of hibernacula offers a powerful method to characterize hibernation timing at the demographic level [19]. Although this technique does not quantify energy reserves or expenditure, the longest time between two registrations during the winter period (referred to here as the ‘longest hibernation period’, or LHP) serves as a useful proxy for overall hibernation timing [19].

Here, we use individualized data of 1132 RFID-tagged bats collected over seven years to document how species identity, sex and age as well as annual variation shape hibernation phenology in two sympatric European bat species. Our two study species, Daubenton’s bat (*Myotis daubentonii*) and Natterer’s bat (*Myotis nattereri*) are similarly sized (Daubenton’s bat: 6–10 g, Natterer’s bat: 7–10 g; [28]), show a high range overlap, and often share the same hibernaculum [28], but differ in their foraging ecology. Daubenton’s bats mainly hunt for aquatic insects found above or on the water surface [28, 29], whose phenology is highly seasonal [30]. Natterer’s bats primarily glean insects from surfaces [28], and as a result are better able to cope with cool, wet weather conditions [31], and feed on inactive prey during the winter [32].

In accordance with the species-specific and demographic differences in hibernation timing outlined above, we expect that: (1) Natterer’s bats will enter later and emerge from underground hibernation earlier than Daubenton’s bats, as they are able to forage during poor weather conditions in autumn and spring (compare [33]), (2) males will enter the hibernaculum later than females, so as to replenish and compensate for depleted fat resources invested into autumn mating activity (compare [34]), whereas (3) females will emerge from hibernation earlier than males due to the advantages of early parturition, (4) juveniles (sensu young-of-the-year) enter later and emerge earlier than adults due to the extra energetic costs of growth and the observation that their fat reserves are often lower than that of adults prior to hibernation [35, 36], and (5) that hibernation phenology might vary among years in both species, if individuals are able to plastically adapt their hibernation phenology in reaction to varying environmental conditions.

## Results

**Fig. 1:**
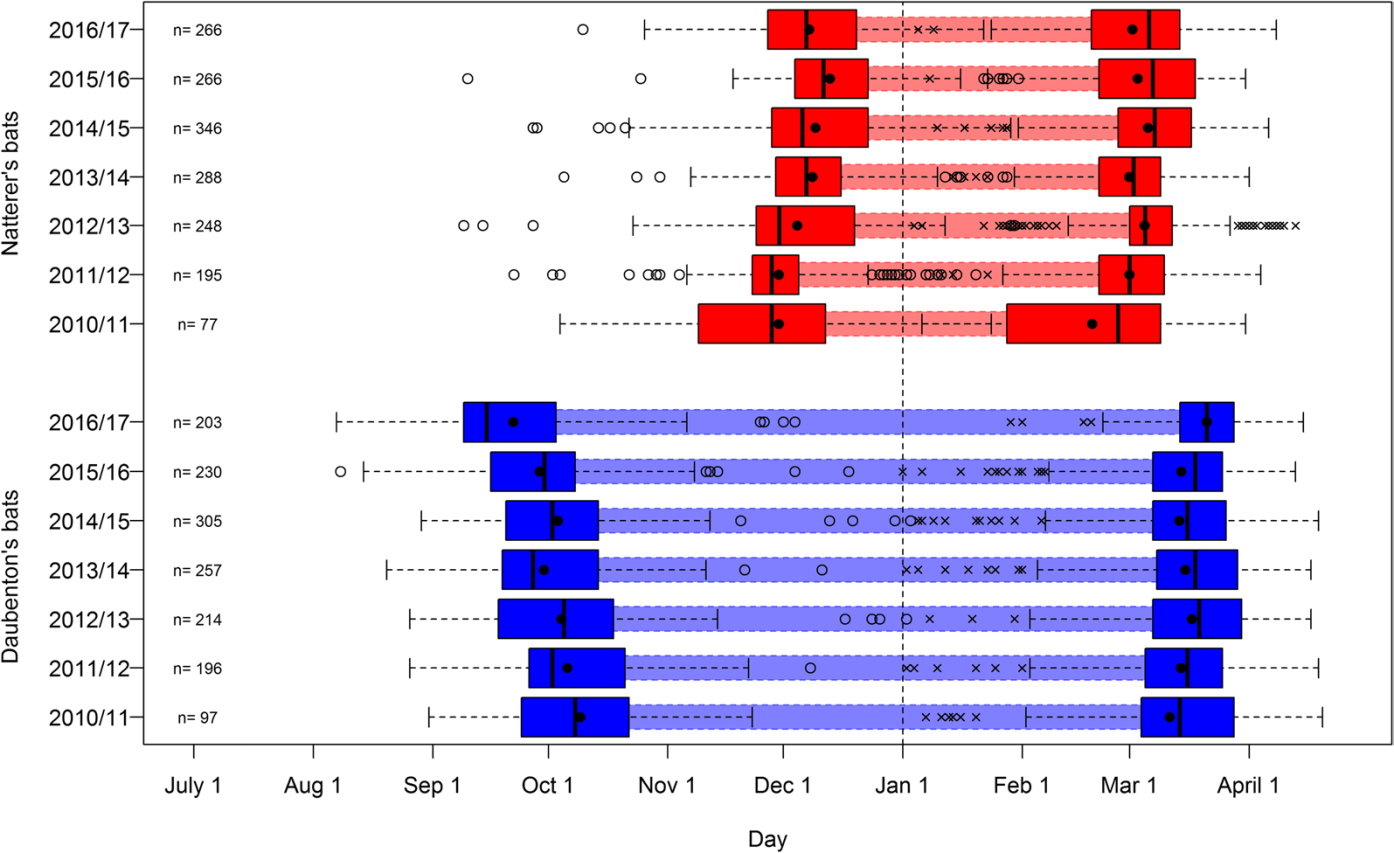
Observed longest hibernation periods per year for Natterer’s bats and Daubenton’s bats. Horizontal boxplots denote entrance and emergence dates (vertical line: median; filled circle: mean; open circles: entrance date outliers; x: emergence date outliers); the period spent inside the hibernaculum is denoted by the shaded area between boxplots. Corresponding calender dates of entrance, emergence and duration of the longest hibernation periods per year are provided in Additional file 1

We analyzed 1502 LHPs (longest hibernation period: the longest time between two registrations during the winter period) of 539 individual Daubenton’s bats and 1686 LHPs of 593 individual Natterer’s bats during seven winter periods (see Table 1 for sample sizes per year). In Daubenton’s bats, median LHP duration ranged from the mid September to the third week of March. In contrast, the median LHP duration in Natterer’s bats started about two and a half months later, in early December, and ended up to a half month earlier, in early March. With a total duration of approximately six months, LHP durations (median) in Daubenton’s bats were about twice as long as in Natterer’s bats (Fig. 1, Additional file 1).

**Table 1:** Sample size of all analyzed individuals per species, sex, age and winter period

Winter period	*Myotis daubentonii* (n = 539)				*Myotis nattereri* (n = 593)			
	Male		Female		Male		Female	
	ad	juv	ad	juv	ad	juv	ad	juv
2010/11	17	15	48	17	13	15	38	11
2011/12	72	21	86	17	50	42	78	25
2012/13	103	7	93	11	83	29	115	21
2013/14	120	22	102	13	105	42	124	17
2014/15	159	15	113	18	141	20	165	20
2015/16	141	0	89	0	128	0	138	0
2016/17	108	13	75	7	121	23	113	9
Sum	720	93	606	83	641	171	771	103
	1502				1686			

Number of marked individuals that were included in the model for each winter period (ad = adult; juv = juvenile)

### Effects of sex, age and winter period on the timing of hibernation

**Fig. 2:**
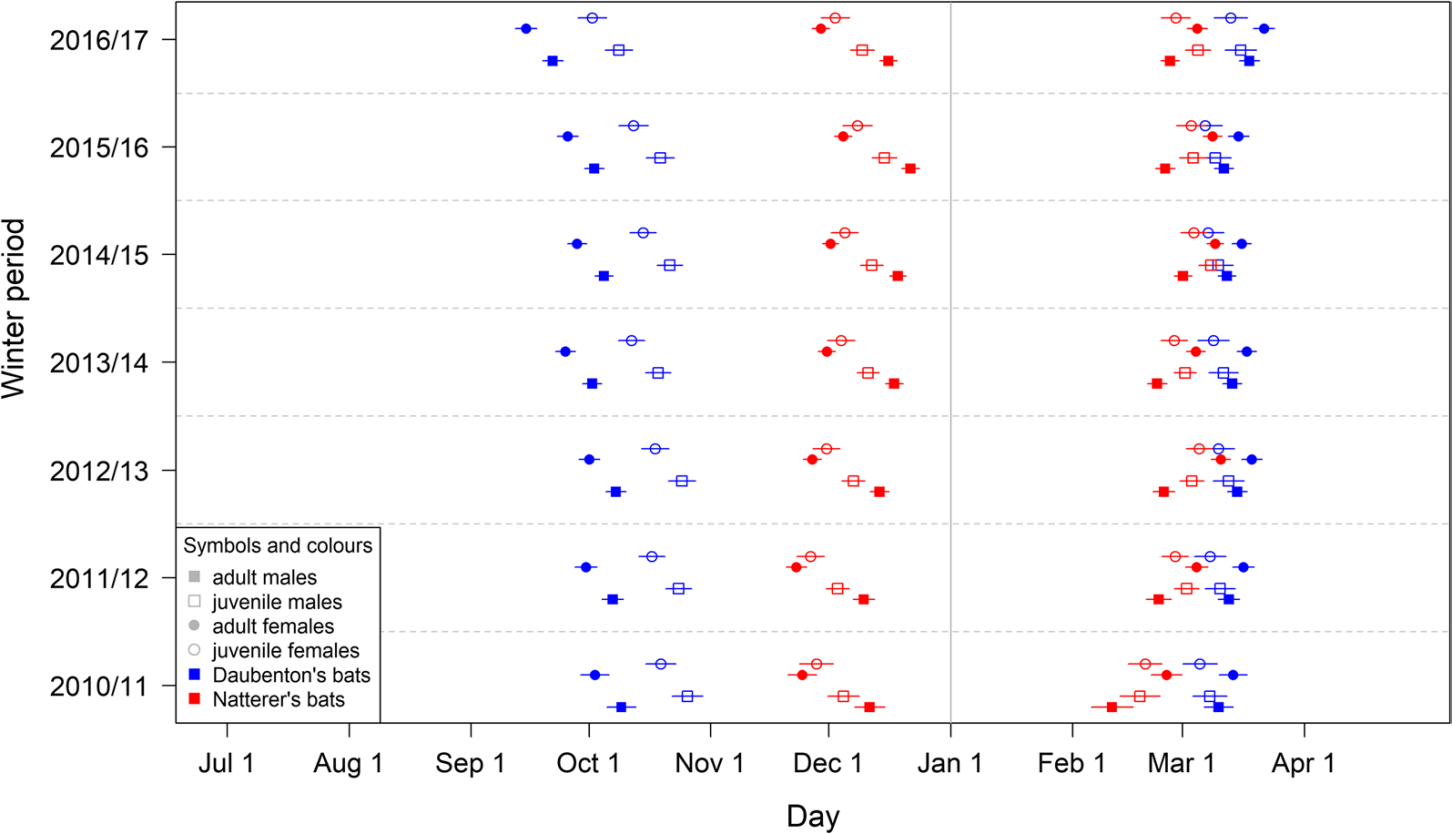
Estimated entrance and emergence of longest hibernation periods for both study species. Estimates of the four species-specific best fitted models of LHP timing (entrance, emergence) for Natterer’s bats (red) and Daubenton’s bats (blue). Sex is denoted by symbol shape (male = square, female = circle), and age by filling (adult = filled, juvenile = empty)

All tested parameters, “sex”, “age class” and “winter period”, improved the models for the three response variables “entrance”, “emergence” and “duration” of LHP, but in a species-specific way (Tables 2 and 3). In Natterer’s bats, sex was the strongest predictor of LHP, whereas in Daubenton’s bats age was more important. In both species, winter period influenced LHPs, indicating that hibernation phenology differed among years (Fig. 2, Additional file 1). For Natterer’s bats, all selected best models included an interaction between sex and age, whereas in Daubenton’s bats this interaction only considerably improved the hibernation emergence model. An additional interaction between sex and winter period considerably improved the LHP emergence model in Natterer’s bats, whereas in Daubenton’s bats it only slightly improved the model, but did not exceed the threshold of dAICs of 2 (Table 2). Visual inspection of residual plots confirmed that the best models for the entrance and duration of LHP fulfilled the assumption of a uniform distribution for both species. However, the best models for the LHP emergence did not. This suggests that the latter models lack one or more additional explanatory variable(s) that may have a significant influence on the response variable “LHP emergence”. Female pre-hibernation body mass [19, 37] has been found to influence hibernation phenology. Thus, this parameter may constitute an important missing explanatory variable in our hibernation emergence models. On an individual level, annual timing of LHP entrance, emergence and duration showed moderate repeatability (R_ad_ = 0.3–0.42; Table 3).

**Table 2:** Top two models for entrance, emergence and duration of the longest hibernation period

Model	K	AIC	dAIC
*Daubenton*’*s bat LHP entrance*			
**Sex + age + winter period**	**11**	**12,633.31**	
Sex + age + winter period + interaction age-sex	12	12,635.15	1.85
*Natterer*’*s bat LHP entrance*			
**Sex + age + winter period + interaction age-sex**	**12**	**14,003.95**	
Sex + age + winter period	11	14,027.34	23.40
*Daubenton*’*s bat LHP emergence*			
**Sex + age + winter period + interaction age-sex**	**12**	**12,593.91**	
Sex + age + winter period + interaction age-sex + interaction sex-winter period	18	12,595.80	0.25
*Natterer*’*s bat LHP emergence*			
**Sex + age + winter period + interaction age-sex + interaction sex-winter period**	**18**	**13,614.58**	
Sex + age + winter period + interaction age-sex	12	13,619.49	4.90
*Daubenton*’*s bat LHP duration*			
Sex + age + winter period + interaction age-sex	12	13,891.07	
**Sex + age + winter period**	**11**	**13,892.53**	**1.46**
*Natterer*’*s bat LHP duration*			
**Sex + age + winter period + interaction age-sex**	**12**	**14,727.99**	
Sex + age + winter period	11	14,800.87	72.88

The best two models for Daubenton’s bats and Natterer’s bats shown here are based on model selection for the best fitted fixed effect structure. In all cases, best models (bold) were selected with regard to the effect of sex, age, winter period, and the interaction between age and sex, with individual ID included as a random effect. For the model of LHP emergence, the interaction between sex and winter period was additionally considered. Model selection was based on the AIC, and we used a threshold of dAIC of > 2 for a more complex model to be selected over a simpler one

**Table 3:** Estimates for entrance, emergence and duration of the longest hibernation period for (A) Daubenton’s bat, and (B) Natterer’s bat

(A) Daubenton’s bat (number of observations: 1502 of 539 individuals)			
Model	LHP entrance ~ sex + age + wp + (1 | transp)	LHP emergence ~ sex*age + wp + (1 | transp)	LHP duration ~ sex + age + wp + (1 | transp)
Fixed effects	Estimates (95% CI)		
Intercept	101.2 (97.6;104.9)	253.2 (249.6;256.8)	152.5 (147.0;158.1)
Sex f (Ref = m)	− 6.8 (− 9.3;− 4.3)	3.7 (1.2;6.2)	9.4 (5.7;13.1)
Age juv (Ref = ad)	16.8 (14.2;19.5)	− 2.2 (− 5.7;1.3)	− 22.2 (− 26.2;− 18.2)
wp 11/12 (Ref = 10/11)	− 2.2 (− 5.8;1.3)	2.6 (− 1.0;6.2)	4.9 (− 0.6;10.3)
wp 12/13 (Ref = 10/11)	− 1.4 (− 5.1;2.3)	4.7 (1.1;8.4)	6.1 (0.4;11.7)
wp 13/14 (Ref = 10/11)	− 7.4 (− 11.1;− 3.8)	3.5 (− 0.2;7.1)	10.9 (5.4;16.5)
wp 14/15 (Ref = 10/11)	− 4.5 (− 8.1;− 0.9)	2.1 (− 1.5;5.7)	6.5 (1.0;12.0)
wp 15/16 (Ref = 10/11)	− 6.9 (− 10.7;− 3.1)	1.4 (− 2.4;5.2)	8.18 (2.3;13.9)
wp 16/17 (Ref = 10/11)	− 17.5 (− 21.4;− 13.6)	7.8 (4.0;11.7)	25.1 (19.2;30.9)
Sex*age f.juv (Ref = m.ad)		− 6.3 (− 11.2;− 1.3)	
Random intercept SD (95% CI)	11.5 (10.3;12.6)	10.4 (9.0;11.8),	16.8 (14.9;18.7)
Residual SD (95% CI)	13.5 (12.8;14.0)	13.6 (12.9;14.2)	20.7 (19.7;21.6)
R_ad_ (95% CI)	0.42 (0.37;0.48)	0.37 (0.32;0.43)	0.40 (0.34;0.46)

(B) Natterer’s bat (number of observations: 1686 of 593 individuals)			
Model	LHP entrance ~ sex * age + wp + (1 | transp)	LHP emergence ~ sex * age + wp + wp:sex + (1 | transp)	LHP duration ~ sex * age + wp + (1 | transp)
Fixed effects	Estimates (95% CI)		
Intercept	164.4 (160.6;168.1)	226.1 (220.9;231.3)	64.7 (60.0;69.3)
Sex f (Ref = m)	− 17.1 (− 19.3;− 14.9)	13.9 (7.4;20.3)	26.8 (23.7;29.6)
Age juv (Ref = ad)	− 6.7 (− 9.3;− 4.0)	7.1 (4.7;9.6)	13.0 (9.8;16.2)
wp 11/12 (Ref = 10/11)	− 1.5 (− 5.2;2.3)	11.9 (6.6;17.2)	10.6 (6.1;15.1)
wp 12/13 (Ref = 10/11)	2.5 (− 1.2;6.2)	13.2 (7.9;18.5)	10.7 (6.2;15.1)
wp 13/14 (Ref = 10/11)	6.2 (2.6;9.9)	11.5 (6.2;16.8)	2.4 (− 2.0;6.9)
wp 14/15 (Ref = 10/11)	7.2 (3.5;10.9)	18.0 (12.6;23.4)	7.2 (2.7;11.7)
wp 15/16 (Ref = 10/11)	10.4 (6.6;14.2)	13.5 (8.0;19.1)	1.5 (− 3.2;6.1)
wp 16/17 (Ref = 10/11)	4.8 (1.0;8.6)	14.8 (9.3;20.2)	5.1 (1.2;10.5)
Sex*age f.juv (Ref = m.ad)	10.3 (6.3;14.3)	− 12.6 (− 16.3;− 8.8)	− 21.6 (− 26.5;− 16.8)
Sex*wp 11/12 (Ref = m.10/11)		− 4.2 (− 11.0;2.5)	
Sex*wp 12/13 (Ref = m.10/11)		0.6 (− 6.1;7.3)	
Sex*wp 13/14 (Ref = m.10/11)		− 4.0 (− 10.8;2.7)	
Sex*wp 14/15 (Ref = m.10/11)		− 5.6 (− 12.4;1.1)	
Sex*wp 15/16 (Ref = m.10/11)		− 1.8 (− 8.8;5.2)	
Sex*wp 16/17 (Ref = m.10/11)		− 7.0 (− 13.9;− 0.01)	
Random intercept SD (95% CI)	8.8 (7.8;9.9)	9.0 (8.1;9.9)	13.4 (12.1;14.7)
Residual SD (95% CI)	13.4 (12.9;14.0)	11.6 (11.1;12.0)	15.9 (15.2;16.5)
R_ad_ (95% CI)	0.30 (0.25;0.36)	0.38 (0.33;0.44)	0.42 (0.37;0.48)

For each species model results for the three variables (entrance, emergence, duration) are reported for the model with the best fixed effect structure (see Table 2; wp = winter period). A random intercept controls for repeated observations of the same individual across years (transponder = transp). Estimates were obtained by REML. Parentheses denote the 95% confidence interval (CI) of each estimate. For the fixed effects the reference levels (Ref) are mentioned in parentheses (f = female, m = male; ad = adult, juv = juvenile). For each model, the resulting intra-individual correlation values (R_ad_ = adjusted repeatability) are given in the final row

In Daubenton’s bats, estimated LHP dates confirmed that adult females were the first to enter and last to emerge, and thus had the longest LHPs. Adult males typically entered into the LHP a week later than females, and emerged earlier, resulting in a 10-day shorter overall hibernation duration than in adult females. Juveniles also entered their LHP onsets later than adult females, with juvenile females LHPs entering around a week earlier than juvenile males. According to our models, juvenile females emerged from their LHPs as the earliest intraspecific group, around two or three days earlier than juvenile males.

In Natterer’s bats, our models likewise found that adult females were the first to enter and last to emerge and had the longest intraspecific LHPs. Adult male entry was substantially later, and emergence was up to two weeks earlier, resulting in the shortest intra- and interspecific LHP (27 days shorter than in adult females). Juvenile Natterer’s bats entered their LHP later than adult females, but earlier than adult males, and emerged earlier than adult females but later than adult males. Moreover, juvenile females entered into their LHPs one week earlier than juvenile males. Altogether, juvenile male Natterer’s bats had shorter LHPs than juvenile females, both having shorter LHPs than adult females, but unlike in Daubenton’s bats, longer LHPs than adult males.

Estimated hibernation entry varied among the seven analyzed winter periods by up to 17 days in Daubenton’s bats, and emergence by 18 days (males) and 14 days (females) in Natterer’s bats. Variation between the shortest and longest estimated annual mean LHP duration was much larger for Daubenton’s bats than for Natterer’s bats (25 days vs. 10 days). Interestingly there was one outlier year: The winter period 2010/2011 contained the earliest estimated LHP emergence dates and the shortest LHP durations for both species. In Daubenton’s bats, entrance was also latest in this winter period.

Notably, according to our model, Daubenton’s bats entered their LHPs progressively earlier during all following autumns. In contrast, the Natterer’s bats model usually revealed later LHP entrance dates than in 2010, except for 2011 (Fig. 2, Additional file 1).

## Discussion

We investigated the individual hibernation phenology in two common sympatric temperate-zone bat species over multiple years using RFID-tags to quantify the longest period concurrently spent within a hibernaculum. We observed marked differences in overall hibernation duration between species, with Daubenton’s bats hibernating up to twice as long as Natterer’s bats. Linear mixed models found that sex was the strongest predictor of hibernation phenology in Natterer’s bats, whereas in Daubenton’s bats age was more important. Nevertheless, the effects of sex and age were similar in both species; adult females had the longest hibernation duration, and juveniles generally had shorter hibernation durations than adults, with the exception of adult male Natterer’s bats, which had the shortest overall duration. Finally, timing of all parameters varied over years, with Daubenton’s bats showing a conspicuous progressive regression in hibernation entry, potentially indicating an influence of changing environmental conditions on hibernation phenology.

The timing of hibernation phenology has important consequences for an individual’s survival and reproductive success. The importance of minimizing the ecological and physiological costs of hibernation have recently been highlighted as an important driver that may favor reduced expression of hibernation when possible (see optimal hibernation theory; [3]). Characterizing the entrance and emergence phenology across demographic classes within and between species allows for an understanding of the broader pressures shaping hibernation behavior. In this context, several of the differences observed between the two species investigated here may point to more general patterns driving hibernation phenology across fat-storing hibernators.

### Species effects

The observed differences in hibernation phenology between our two study species are likely a reflection of their different foraging niches, as the experienced local weather conditions were identical and their body size, and thus their fat storage capacity and energy metabolism, are broadly comparable. Daubenton’s bats primarily forage on aquatic insects [29], whose emergence starts in April, peaks in August, and terminates in October [30], closely matching the hibernation phenology observed here. In contrast, the gleaning abilities of Natterer’s bats allow them to exploit inactive prey [32], allowing them to efficiently hunt at lower temperatures, thereby potentially explaining their much shorter overall hibernation duration. Previous studies have similarly highlighted the role of species-specific foraging niches and food availability in shaping the timing of reproduction in sympatric *Myotis* species [31, 38]. Nevertheless, when compared to the hibernation phenology of *M. lucifugus* in central Canada [19], it is clear that broader extrinsic factors such as climate also play a large role. In this species, mean hibernation duration in all sex/age classes was over 230 days, roughly three times longer than observed for Natterer’s bats in this study.

The far shorter overall duration of hibernation may allow Natterer’s bats to reduce the ecological and physiological costs of hibernation on the whole, but may also increase their susceptibility to variations in prey availability and climate instability. A clear example of this is the winter-period 2010/2011, during which there was an unusually heavy snowfall and a long-lasting snow cover from the second half of November onwards. Reusch et al. [39] observed a survival rate of only 38% in Natterer’s bats in our study population during this winter (compared to over 80% in other years), suggesting many individuals were forced to enter hibernation before their energy resources were sufficiently high to ensure a successful hibernation. More broadly, winter survival rates of Natterer’s bats were consistently lower than of Daubenton’s bats, but summer survival rates were higher [39], potentially suggesting that the theoretical ability to feed throughout longer periods of the year does not mean that it is necessarily advantageous to do so, or that the increased mortality during winter is compensated by higher survival during summer due to the avoided physiological costs of hibernation.

### Sex effects

The observed sex differences in hibernation phenology generally corresponded to predicted effects of the desynchronization of reproductive investment between males and females seen in temperate-zone bats [19], with the exception that males not only entered hibernation later but also emerged from hibernation earlier than females. Males invest heavily in spermatogenesis and mating behavior immediately prior to hibernation. Indeed, Kohyt et al. [33] found that the seasonal and nightly body condition of adult male Natterer’s bats only increased after the species swarming activity peak in early October. This suggests that their special foraging ability enables the males to postpone the accumulation of fat reserves until just before entering the hibernaculum. Unlike in *M. lucifugus* [19], adult males emerged from the hibernaculum earlier than adult females in both study species. By strongly investing in mating activities in autumn, it is conceivable that male bats may be forced to emerge from hibernation early because of depleted fat reserves [34]. Males may be able to compensate for reduced foraging success in spring by using daily torpor as a strategy to avoid bad feeding conditions [40]. In contrast, females pay a cost of reduced fetal growth if they use daily torpor in spring [41]. As a result, if spring foraging success is unpredictable, it may be that delaying the departure from the hibernaculum until feeding conditions are more stable is favorable for females despite the advantages of early parturition.

### Age effects

Both delayed entry and early emergence from the hibernaculum in juveniles, may be caused by a large energetic investment into growth and a slower accumulation of sufficient energy reserves prior to hibernation [36, 42]. In addition, in both of our study species, juvenile males may already invest in reproduction in their first year [43, 44], suggesting an increased energy investment of juvenile males relative to juvenile females. This may explain why juvenile males entered the hibernaculum considerably later than juvenile females. This strategy may however come with a considerable trade-off when a species hibernation onset is already generally very late and may explain why juvenile male Natterer’s bats have the lowest winter survival rate of all sex/age classes [45]. Age related differences in emergence were considerably less pronounced, suggesting that increased investment in filling up energy reserves prior to hibernation is preferable to early emergence. As in the observed later emergence of adult females, this may be related to the unpredictability of foraging success in spring. In *M. lucifugus* and *Myotis volans*, Schowalter [35] found that juveniles were active later than adults, although Norquay and Willis [19] did not observe a difference. However, they did find that juvenile female *Myotis lucifugus* emerged from hibernation significantly later than adult females [19], likely emphasizing the importance of early emergence for early parturition in the species where females do not reproduce in their first year.

### Annual variation in hibernation phenology

We detected shifts in yearly hibernation phenology in both species, suggesting some ability to flexibly time hibernation, depending on yearly environmental conditions. Zervanos et al. [46] observed differences in hibernation timing along a latitudinal gradient for woodchucks (*Marmota monax*) suggesting phenotypic plasticity allowing them to adjust energy use in response to different climatic conditions. However, other factors may limit plasticity in hibernation phenology based on environmental conditions alone as seen in Edible dormice (*Glis glis*), where individuals entered hibernation despite favorable feeding conditions presumably as a result of increased predation risk during autumn [37].

How these effects interact to shape hibernation timing have important consequences for their ability to adapt to climate change. Our findings imply that changing environmental conditions may influence sympatric bat populations divergently (see [20]). Bats with flexible diets such as Natterer’s bats may benefit from the successively extended activity period of some insect species in autumn and early spring [47, 48], and thus reduce their hibernation duration. Despite the potential advantages of such a reduction due to gradual weather changes, the predicted increase in volatility of summer and winter precipitation and storms [49], may strongly impact survival in some years, as seen for Natterer’s bats in 2010/2011 [39]. Daubenton’s bats on the contrary, may be less flexible in exploiting warmer autumn und spring temperature and therefore may be more sensible to potential mismatches with their main food resource. Notably, Chironomidae, an important component of early spring aquatic insect biomass, have been shown to decline with warming temperatures [50], potentially limiting food availability on emergence from hibernation in Daubenton’s bats. Finally, it must be emphasized that in addition to changes in their phenology, species may also adapt with regard to their arousal rates, microclimate selection within the hibernaculum, or social behavior throughout the hibernation period [4–9].

## Conclusion

Taken together, our findings highlight the likely role of foraging niche at a species level and reinforce the importance of timing of reproductive investment between sexes and juvenile development as important drivers of hibernation phenology in temperate-zone bats. These differences may have important evolutionary consequences. Daubenton’s bats spend nearly twice as long underground as Natterer’s bats, and even entered the hibernaculum progressively earlier across the seven years of this study. Further studies are necessary to explore whether this is because prey availability in early autumn is increasing over time and thus individuals are able to accumulate fat stores more quickly, or whether it is out of energy conservation necessity due to a lack of prey availability. In both species the later entry and earlier emergence of males, suggests they are investing heavily in reproductive effort, as is expected for promiscuous species, but also makes them particularly vulnerable and may lead to large differences in mortality and longevity between sexes. Finally, how these phenological differences ultimately affect the incurred physiological costs imposed by hibernation, depend on whether they are associated with differences in torpor bout length or depth. There is good evidence that individuals are able to flexibly adjust these characteristics through microclimate selection and changes in arousal rate (e.g. thrifty female hypothesis; [22]). However further studies are needed to investigate whether the differing phenological and torpor strategies are correlated.

In the context of applied conservation, our results emphasize that the broad generalizations made by current nature conservation legislation and monitoring schemes for hibernation sites and foraging areas likely only effectively protect certain species and demographic groups, and therefore urgently require reconsideration. For example, in Germany human visitation of bat hibernacula is only restricted after October 1st, while our data clearly indicate that most Daubenton’s bats already enter the site before this time and may suffer from disturbance early in their hibernation period [51]. In contrast, given the short hibernation duration in Natterer’s bats, surveys identifying key foraging habitats and tree roosts should also be carried out in late autumn and early spring, and not only during summer [52]. Finally, our results highlight the utility of long-term monitoring of individually marked populations to monitor changes in hibernation phenology and the response of bats to environmental change.

## Methods

### Study area and study population

The studied hibernaculum is a 60 m deep well with a diameter of about two meters, situated inside a small well house, which is located in northwest North Rhine-Westphalia, Germany. In winter, the temperature inside the well ranges from about 2 to 6 °C [34]. Bats enter the well house through a window. To enter the well itself, the bats need to crawl through two small entrances in the well’s wooden lid. Several thousand bats belonging to seven different species are estimated to hibernate in the well each year, most of them being Daubenton’s bats and Natterer’s bats [34].

### Automatic monitoring and bat capture

Since 2010, RFID-transponder readers (EUR-8100 until 2015, LID 650 since then; EURO ID, Germany), with loop antennas of 5 cm height and 8 cm width, continuously recorded the identity, date and time of tagged bats crawling through the well’s entrances. Since there was only one antenna per entrance, we do not have precise information regarding the directionality of the reading events.

Each year since 2009, with the exception of 2015, we captured bats with a harp trap in front of the window inside the well house once per week during August and September. Captured bats were sexed and age was determined as either adult or juvenile (sensu young-of-year; for details see [39]). Each year, up to 150 individuals per species were tagged with an RFID-transponder (Trovan, 100 ID).

### Data sample, organization and time definitions

**Fig. 3:**
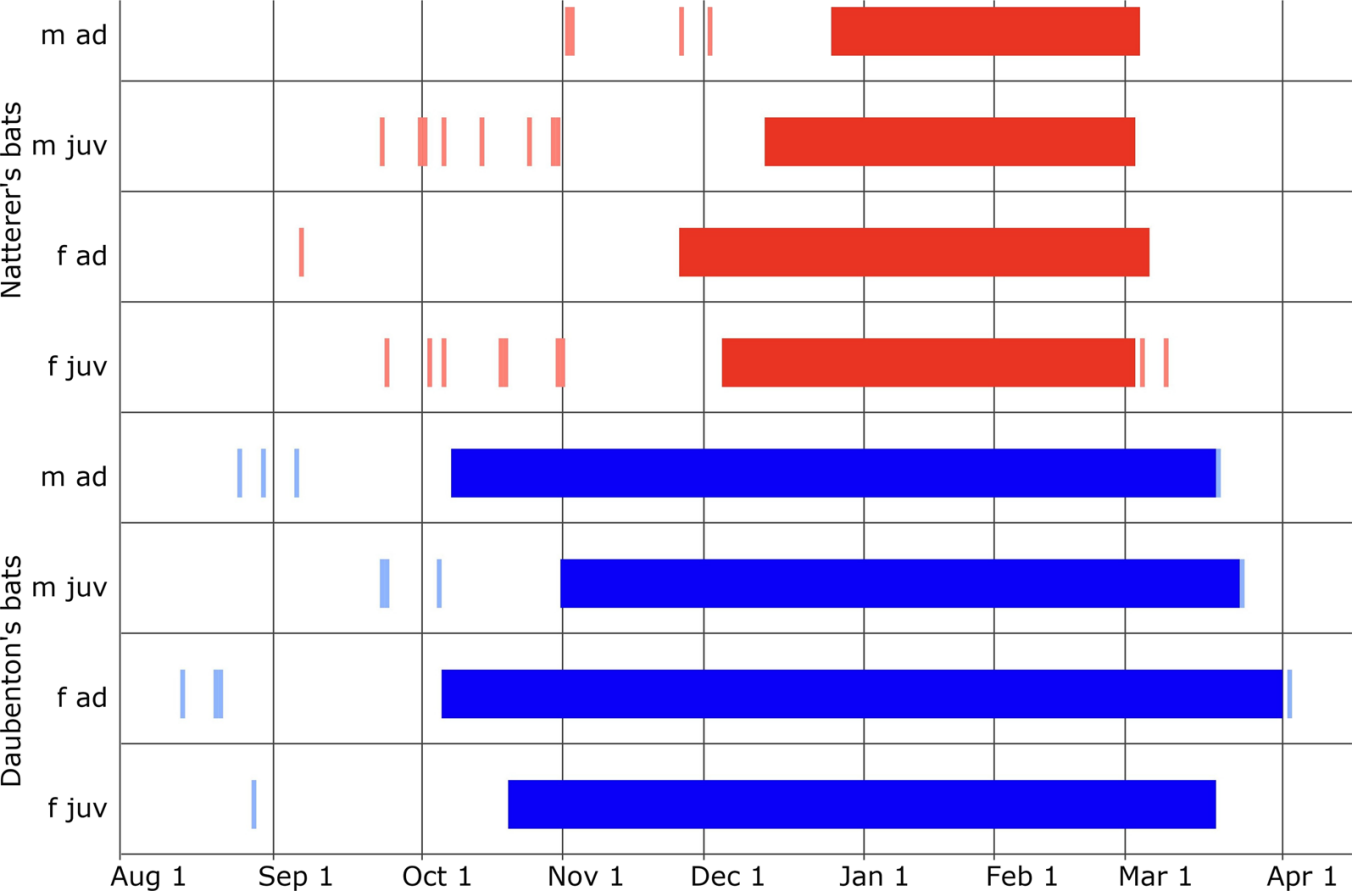
Examples for visual inspections of our longest hibernation period for both study species. Full shaded horizontal bars denote the duration of the LHP, the beginning of the bar the entrance, the end of the bar the emergence (m ad = adult male, m juv = juvenile male, f ad = adult female, f juv = juvenile female). Partially shaded bars outside of the LHP represent daily recordings of the bat individual before and after the longest hibernation period (LHP)

For a first visual inspection of individual activity patterns, we reduced recordings to presence/absence information per day. A bat day ranged from noon of one day to noon of the next day to account for the nocturnal activity of the bats. Next, we visualized every day with recorded activity per individual along a timeline. These figures revealed a behavioral pattern consistent with the known hibernation phenology of bats [28], with up to several daily recordings in late summer and autumn (swarming period), followed by a long time without recordings during the winter (hibernation), after which one or a few recordings were detected again in spring, indicating that the bats had finally left their hibernaculum (Fig. 3).

To quantify hibernation entrance, emergence and duration, we defined the longest time without any recordings of the respective individual within a predefined “winter period” (between 1 July and 30 April) as the “longest hibernation period” (LHP). We defined the last recording of an individual before the LHP as their entry. Accordingly, its first recording after the longest time without detected activity was defined as their emergence. We applied a filter to exclude all LHPs with entrance dates after 1 February or emergence dates before 31 December to filter out instances of mortality during the winter period (leaving the LHP with an entrance and emergence date during the swarming phase), and failure of the tag or recording devices. Overall, 15% of our total dataset on individual LHPs were excluded from the analysis by our filter criteria, which broadly corresponds to the mortality estimates for this population [39] and is even well below the mortality estimates for other populations [45]. This suggests that our filter worked well for our data set, and did not remove many individuals that emerged briefly between two or more extended hibernation phases.

### Data analysis

To test for differences in the mean entrance, emergence and duration of the LHP between species, we considered the seven winter periods (2010/2011–2016/2017) separately. To identify the suitable statistical test, we used a Levene’s test to test for variance of homogeneity across winter periods. Since the variance was not homogenous, we applied a Games Howell test with different sample sizes per group and year (Table 1 and Additional file 2).

Based on the significant differences (p < 0.001) in the aforementioned hibernation parameters between the species in every winter period, we applied a separate restricted maximum likelihood linear mixed-effect model per species. Furthermore, based on absence of strong correlations (Kendall’s tau coefficient, value < 0.70, see [53]) between entrance, emergence and duration within species, we additionally decided to apply separate models to each of the three parameters, resulting in six total models. All models accounted for the repeated observations per individual by including an individual-specific random intercept.

To test for differences in hibernation phenology between males and females, and adults and juveniles, sex and age class were included as binary fixed effects. Furthermore, we investigated the possibility of sex-dependent age effects by including a corresponding interaction in all model selection processes. Winter period was included as a proxy for yearly environmental variation into the model selection process. Additionally, we considered an interaction between sex and winter period in the modeling process of LHP emergence to account for sex-specific differences in their behavior with respect to different winter periods. We compared models based on AIC and selected simpler models whenever dAIC_i_< 2 (dAIC_i_ = AIC_i_ – AIC_min_ [54]. All models were fit in R, version 3.4.0 (library lme4, function lmer, gaussian family). To control for the quality of the chosen best models, we visually analyzed whether residual plots were fulfilling the assumption of a uniform distribution.

Furthermore, we calculated the adjusted repeatability (R_ad_) using the R-package rptR as the proportion of variance explained by the between-individual differences of the random intercept adjusting for the fixed effects [55, 56]. The 95% confidence intervals of R_ad_ were estimated based on 1000 bootstrapping runs and 1000 permutations [56].

## Supplementary Information

**Additional file 1: Table S1.** Provides a summary of observed LHP entrance dates, emergence dates and durations. **Table S2.** Provides calender dates of estimated LHP entrance and emergence and durations as well as ranges between minimum and maximum values during our seven year study period.

**Additional file 2: Figures S1–S3.** Provides boxplots of observed LHP entrance (1), emergance (2) and duration (3) split per year and significance levels resulting from the Games-Howell test.

**Additional file 3. **Raw data for: F. Meier, L. Grosche, C. Reusch, V. Runkel, J. van Schaik and G. Kerth. BMC Ecology and Evolution, 2022. Long-term individualized monitoring of sympatric bat species reveals distinct species- and demographic differences in hibernation phenology.

## Data Availability

All raw entrance and emergence data used in this paper are found in Additional file 3.
