# Supplementary material for: Long-term individualized monitoring of sympatric bat species reveals distinct species- and demographic differences in hibernation phenology
Source: BMC Ecol Evol. 2022 Jan 28;22:7. doi: 10.1186/s12862-022-01962-6 (PMC8796590; doi:10.1186/s12862-022-01962-6)
Supplement: Supplementary file 1 — Additional file 1: Table S1. Provides a summary of observed LHP entrance dates, emergence dates and durations. Table S2. Provides calender dates of estimated LHP entrance and emergence and durations as well as ranges between minimum and maximum values during our seven year study period. [file 12862_2022_1962_MOESM1_ESM.docx]

**Additional File 1**

**Main manuscript (Meier et al. 2022):** **Long-term individualized monitoring of sympatric bat species reveals distinct species- and demographic differences in hibernation phenology.** **BMC Ecology and Evolution**

**Table S1** provides a summary of observed LHP entrance dates, emergence dates and durations. **Table S2** provides calender dates of estimated LHP entrance and emergence and durations as well as ranges between minimum and maximum values during our seven year study period.

**Table S1:** Observed LHP entrance dates, emergence dates and durations.

|  | LHP entrance | | LHP emergence | | LHP duration | |
| --- | --- | --- | --- | --- | --- | --- |
|  | Md | Mn | Md | Mn | Md | Mn |
|  | min/max values during study period 2010/11 - 2016/17 | | | | | |
| Min | 08 Aug/31 Aug | 09 Sept/05 Okt | 01Jan/29 Jan | 04 Jan/14 Jan | 18/77 | 15/34 |
| 1st Qu | 09 Sept/26 Sept | 09 Nov/04 Dec | 04 Mar/14 Mar | 28 Jan/01 Mar | 132/163 | 56/79 |
| Med | 15 Sept/08 Okt | 28 Nov/12 Dec | 14 Mar/21 Mar | 26 Feb/08 Mar | 157/185 | 81/95 |
| 3rd Qu | 02 Okt/21 Okt | 02 Dec/23 Dec | 25 Mar/30 Mar | 09 Mar/18 Mar | 173/198 | 96/103 |
| Max | 23 Nov/02 Jan | 22 Jan/31 Jan | 13 Apr/20 Apr | 31 Mar/13 Apr | 216/227 | 153/198 |

Only the minimum and maximum values during the seven-year study period between 2010/11 and 2016/17 are given. Recorded values for the two study species Daubenton`s bats (Md) and Natterer`s bats (Mn) are given as median (Med), 1st quartile (1st Qu), 3rd quartile (3rd Qu), minimum (Min) and maximum (Max). For yearly sample sizes see Table 1.

**Table S2:** Estimated LHP entrance and emergence calendar dates and LHP durations in days for Daubenton`s bats and Natterer`s bats for our seven winter periods.

| winter period | *Myotis daubentonii* | | | | *Myotis nattereri* | | | |
| --- | --- | --- | --- | --- | --- | --- | --- | --- |
|  | m ad | m juv | f ad | f juv | m ad | m juv | f ad | f juv |
| LHP entrance | | | | | | | | |
| 2010/11 | 9 Oct | 26 Oct | 2 Oct | 19 Oct | 11 Dec | 5 Dec | 24 Nov | 28 Nov |
| 2011/12 | 7 Oct | 24 Oct | 30 Sept | 17 Oct | 10 Dec | 3 Dec | 23 Nov | 26 Nov |
| 2012/13 | 8 Oct | 25 Oct | 1 Oct | 18 Oct | 14 Dec | 7 Dec | 27 Nov | 30 Nov |
| 2013/14 | 2 Oct | 19 Oct | 25 Sept | 12 Oct | 18 Dec | 11 Dec | 30 Nov | 4 Dec |
| 2014/15 | 5 Oct | 22 Oct | 28 Sept | 15 Oct | 19 Dec | 12 Dec | 1 Dec | 5 Dec |
| 2015/16 | 2 Oct | 19 Oct | 26 Sept | 12 Oct | 22 Dec | 15 Dec | 5 Dec | 8 Dec |
| 2016/17 | 22 Sept | 9 Oct | 15 Sept | 2 Oct | 16 Dec | 9 Dec | 29 Nov | 3 Dec |
| mean | 4 Oct | 21 Oct | 27 Sept | 14 Oct | 16 Dec | 9 Dec | 28 Nov | 2 Dec |
| range | min 2016/17, max 2010/11 (17d) | | | | min 2011/12, max 2015/16 (12d) | | | |
| LHP emergence | | | | | | | | |
| 2010/11 | 10 Mar | 8 Mar | 14 Mar | 5 Mar | 11 Feb | 18 Feb | 25 Feb | 19 Feb |
| 2011/12 | 13 Mar | 11 Mar | 16 Mar | 8 Mar | 23 Feb | 2 Mar | 5 Mar | 27 Feb |
| 2012/13 | 15 Mar | 13 Mar | 19 Mar | 10 Mar | 24 Feb | 3 Mar | 11 Mar | 5 Mar |
| 2013/14 | 14 Mar | 11 Mar | 17 Mar | 9 Mar | 23 Feb | 2 Mar | 4 Mar | 27 Feb |
| 2014/15 | 12 Mar | 10 Mar | 16 Mar | 8 Mar | 1 Mar | 9 Mar | 9 Mar | 4 Mar |
| 2015/16 | 12 Mar | 9 Mar | 15 Mar | 7 Mar | 25 Feb | 4 Mar | 9 Mar | 3 Mar |
| 2016/17 | 18 Mar | 16 Mar | 22 Mar | 13 Mar | 26 Feb | 5 Mar | 5 Mar | 27 Feb |
| mean | 13 Mar | 11 Mar | 17 Mar | 9 Mar | 23 Feb | 2 Mar | 6 Mar | 28 Feb |
| range | min 2010/11, max 2016/17 (8d) | | | | m: min 2010/11, max 2014/15 (18d); f: min 2010/11, max 2012/13 (14d) | | | |
| LHP duration | | | | | | | | |
| 2010/11 | 153 | 130 | 162 | 140 | 65 | 78 | 91 | 83 |
| 2011/12 | 157 | 135 | 167 | 145 | 75 | 88 | 102 | 93 |
| 2012/13 | 159 | 136 | 168 | 146 | 75 | 88 | 102 | 93 |
| 2013/14 | 163 | 141 | 173 | 151 | 67 | 80 | 94 | 85 |
| 2014/15 | 159 | 137 | 168 | 146 | 72 | 85 | 99 | 90 |
| 2015/16 | 161 | 138 | 170 | 148 | 66 | 79 | 93 | 84 |
| 2016/17 | 178 | 155 | 187 | 165 | 70 | 83 | 97 | 88 |
| mean | 161 | 139 | 171 | 149 | 70 | 83 | 97 | 88 |
| range | min 2010/11, max 2016/17 (25d) | | | | min 2010/2011, max 2011/12 and 2012/13 (10d) | | | |

m ad=adult males, m juv=juvenile males, f ad=adult females, f juv=juvenile females. For each variable, the range between estimates across years is given: min=earliest entrance / emergence date or shortest duration, max= latest entrance / emergence date or longest duration, d= difference in days between min and max. Provided ranges are based on unrounded estimates, and therefore may deviate by a day from ranges of reported integer values.
