# Supplementary material for: Long-term individualized monitoring of sympatric bat species reveals distinct species- and demographic differences in hibernation phenology
Source: BMC Ecol Evol. 2022 Jan 28;22:7. doi: 10.1186/s12862-022-01962-6 (PMC8796590; doi:10.1186/s12862-022-01962-6)
Supplement: Supplementary file 2 — Additional file 2: Figures S1–S3. Provides boxplots of observed LHP entrance (1), emergance (2) and duration (3) split per year and significance levels resulting from the Games-Howell test. [file 12862_2022_1962_MOESM2_ESM.docx]

**Additional File 2**

**Main manuscript (Meier et al. 2022):** **Long-term individualized monitoring of sympatric bat species reveals distinct species- and demographic differences in hibernation phenology.** **BMC Ecology and Evolution**

**Figures S1-S3** provide boxplots of observed LHP entrance (1), emergance (2) and duration (3) split per year and significance levels resulting from the Games-Howell test.


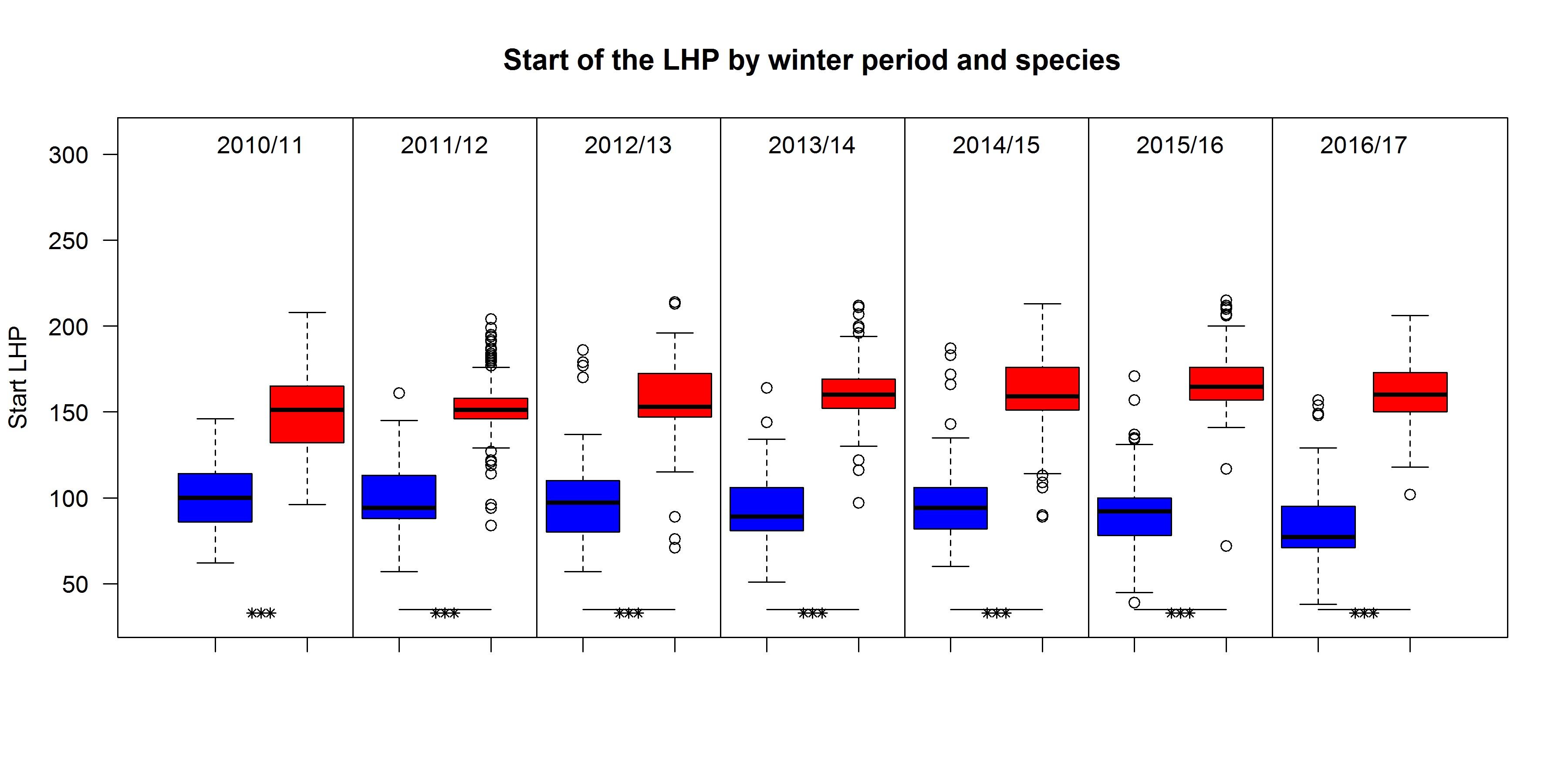


**Figure S1**: Comparison of observed LHP entrance dates (Start LHP) between Daubenton`s bats (md, blue) and Natterer`s bats (mn, red) for each winter period separately. The y-axis shows the entrance date of the LHP (counted days starting from July 1) between July and April of the winter period. On the x-axis years and species can be seen. The stars indicate significance level resulting out of the Games-Howell test (***<0.001, **< 0.01, * <0.05). Sample sizes are: 2010/11: n Md=97, n Mn=77, 2011/12: n Md=196, n Mn=195, 2012/13: n Md=214, n Mn=248, 2013/14:n Md=257, n Mn=288, 2014/15: n Md=305, n Mn=346, 2015/16: n Md=230, n Mn=266, 2016/17: n Md=203, n Mn=266.


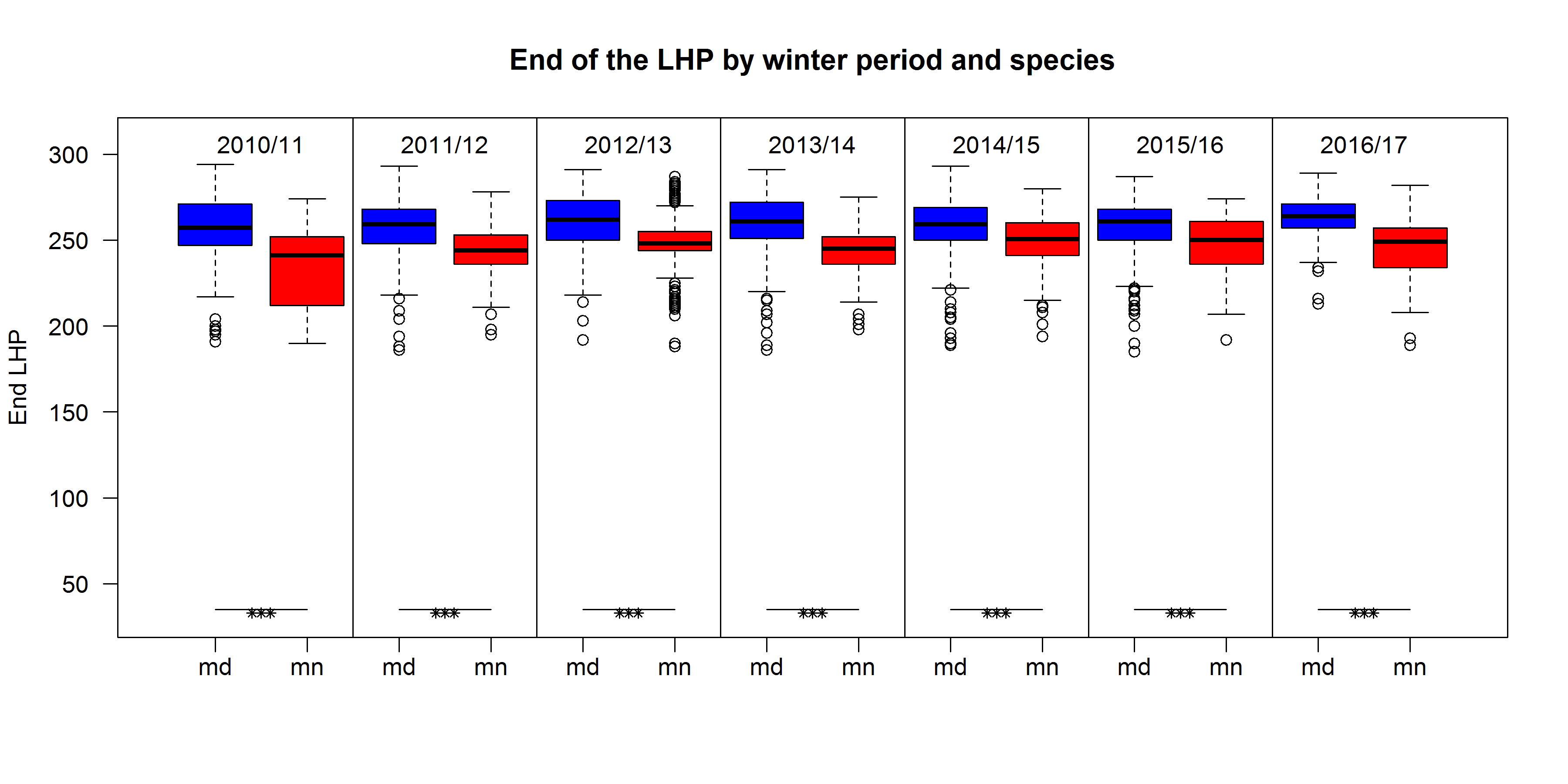


**Figure S2**: Comparison of observed LHP emergence dates (End LHP) between Daubenton`s bats (md, blue) and Natterer`s bats (mn, red) for each winter period separately. The y-axis shows the emergence date of the LHP (counted days starting from July 1) between July and April of the winter period. On the x-axis years and species can be seen. The stars indicate significance level resulting out of the Games-Howell test (***<0.001, **< 0.01, * <0.05). Sample sizes are: 2010/11: n Md=97, n Mn=77, 2011/12: n Md=196, n Mn=195, 2012/13: n Md=214, n Mn=248, 2013/14:n Md=257, n Mn=288, 2014/15: n Md=305, n Mn=346, 2015/16: n Md=230, n Mn=266, 2016/17: n Md=203, n Mn=266.


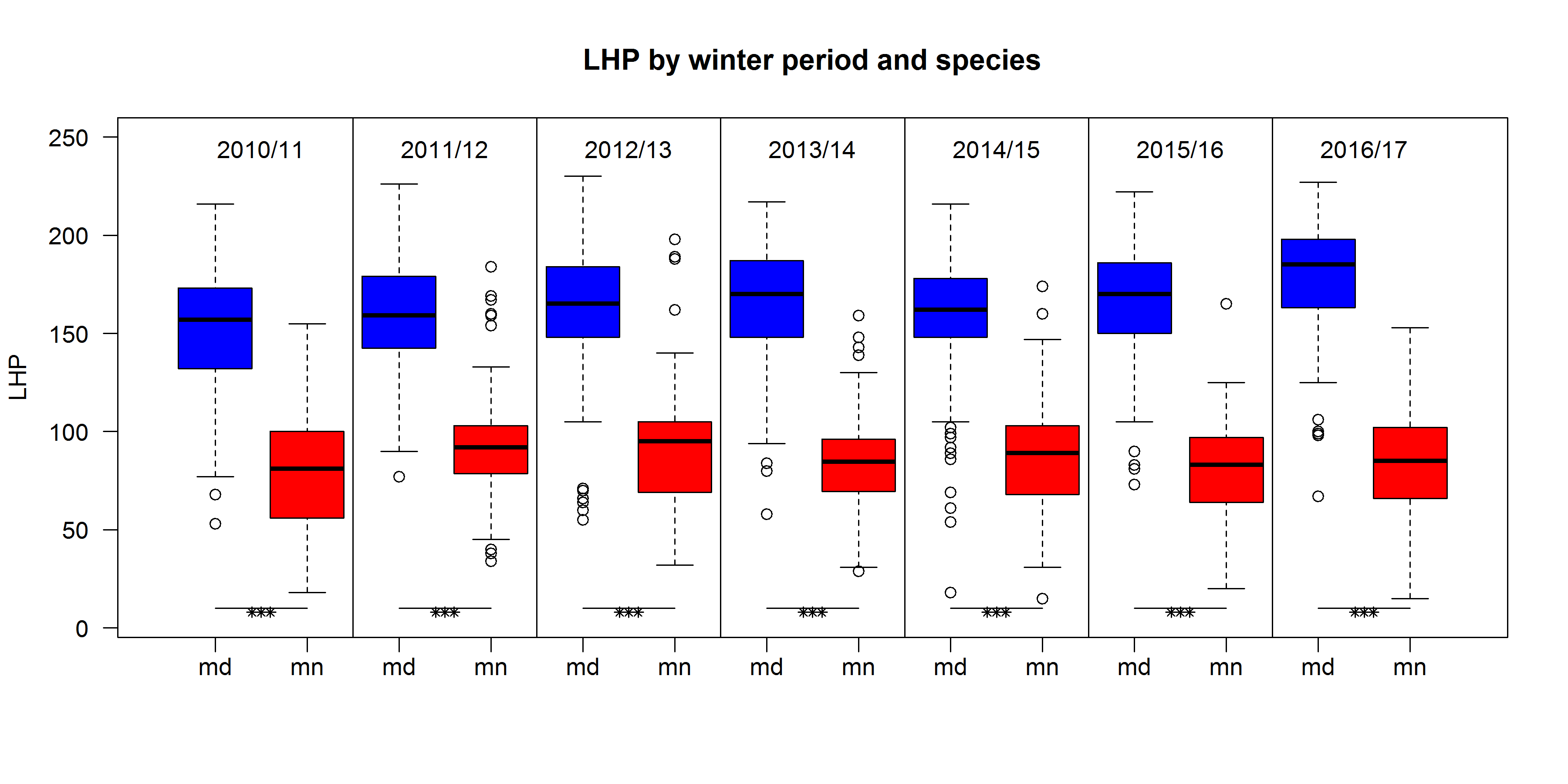


**Figure S3**: Comparison of recorded LHP duration (LHP) between Daubenton`s bats (md, blue) and Natterer`s bats (mn, red) for each winter period separately. The y-axis shows the duration of the LHP in days of the winter period. On the x-axis years and species can be seen. The stars indicate significance level resulting out of the Games-Howell test (***<0.001, **< 0.01, * <0.05). Sample sizes are: 2010/11: n Md=97, n Mn=77, 2011/12: n Md=196, n Mn=195, 2012/13: n Md=214, n Mn=248, 2013/14:n Md=257, n Mn=288, 2014/15: n Md=305, n Mn=346, 2015/16: n Md=230, n Mn=266, 2016/17: n Md=203, n Mn=266.
